# Letter to the Editor Regarding the Impact of ADHD on Outcomes Following Bariatric Surgery: a Systematic Review and Meta-Analysis

**DOI:** 10.1007/s11695-020-05127-6

**Published:** 2020-11-20

**Authors:** Magnus Strømmen, Christian A. Klöckner

**Affiliations:** 1 Centre for Obesity Research, St. Olavs University Hospital, NO-7006 Trondheim, Norway; 2 Department of Clinical and Molecular Medicine, NTNU Norwegian University of Science and Technology, Trondheim, Norway; 3 Department of Psychology, Faculty of Social and Educational Sciences, Norwegian University of Science and Technology, Trondheim, Norway

Obesity Surgery last year published the systematic review and meta-analysis of the impact of ADHD on outcomes following bariatric surgery, authored by Mocanu and colleagues [1]. We read this with great interest, as there is still little evidence on how patients with ADHD benefit from bariatric surgery. When reading the review, we questioned whether one of the included studies actually met the criteria for inclusion. As the review only consisted of five studies, an erroneous inclusion may be significant for the conclusion drawn by the authors.

The study we have in mind is the 2002 paper by Dr. Jules Altfas [2]. The change in BMI reported by Dr. Altfas relates to non-surgical obesity treatment, i.e., diets, counseling, and medication management. Thus, this paper should not have been part of the review. Considering that the decrease in BMI reported by Altfas was surprisingly low to be a result of bariatric surgery, we find it strange that this passed the attention of both the authors and reviewers.

As the patients described in Dr. Altfas’ paper represent a large proportion of the total sample in the meta-analysis, we suggest that Mocanu and colleagues reanalyze the data. In case of a new analysis, we have some comments after reading the original article:

It is not clear what the MINORS score calculated for the five studies has been used for. Has it been used to weight the different studies in the meta-analytical analyses?

Figure 2 does not display mean differences pre-post surgery which is what is indicated in the captions and the text. It displays post-operative BMI for the two groups and then tests the difference between the two. It would also be helpful to get an indication how to read a negative/positive difference here; what is subtracted from what?

It is not clear what the numbers in Figure 3 refer to. Is this week’s follow-up? Why is the difference value between ADHD and non-ADHD here calculated reversed to Figure 2? Taking out Altfas study, the significant difference will disappear.
